# Sirt1 Activity in PBMCs as a Biomarker of Different Heart Failure Phenotypes

**DOI:** 10.3390/biom10111590

**Published:** 2020-11-23

**Authors:** Valeria Conti, Graziamaria Corbi, Maria Vincenza Polito, Michele Ciccarelli, Valentina Manzo, Martina Torsiello, Emanuela De Bellis, Federica D’Auria, Gennaro Vitulano, Federico Piscione, Albino Carrizzo, Paola Di Pietro, Carmine Vecchione, Nicola Ferrara, Amelia Filippelli

**Affiliations:** 1 Department of Medicine, Surgery and Dentistry “Scuola Medica Salernitana”, University of Salerno, 84081 Baronissi, Italy; vconti@unisa.it (V.C.); mvpolito@hotmail.it (M.V.P.); mciccarelli@unisa.it (M.C.); m.torsiello5@studenti.unisa.it (M.T.); e.debellis93@gmail.com (E.D.B.); federica.dauria19@gmail.com (F.D.); vitulanogennaro@yahoo.it (G.V.); fpiscione@unisa.it (F.P.); acarrizzo@unisa.it (A.C.); pdipietro@unisa.it (P.D.P.); cvecchione@unisa.it (C.V.); afilippelli@unisa.it (A.F.); 2 Department of Medicine and Health Sciences, University of Molise, 86100 Campobasso, Italy; graziamaria.corbi@unimol.it; 3 Department of Vascular Physiopathology, IRCCS Neuromed, 86077 Pozzilli, Italy; 4 Department of Translational Medical Sciences, Federico II University of Naples, 80131 Naples, Italy; nicferra@unina.it; 5 Istituti Clinici Scientifici Maugeri SPA-Società Benefit, IRCCS, 82037 Telese Terme (BN), Italy

**Keywords:** heart failure with preserved ejection fraction, heart failure with mid-range ejection fraction, heart failure with reduced ejection fraction, sirtuins, Sirt1

## Abstract

Heart Failure (HF) is a syndrome, which implies the existence of different phenotypes. The new categorization includes patients with preserved ejection fraction (HFpEF), mid-range EF (HFmrEF), and reduced EF (HFrEF) but the molecular mechanisms involved in these HF phenotypes have not yet been exhaustively investigated. Sirt1 plays a crucial role in biological processes strongly related to HF. This study aimed to evaluate whether Sirt1 activity was correlated with EF and other parameters in HFpEF, HFmrEF, and HFrEF. Seventy patients, HFpEF (*n* = 23), HFmrEF (*n* = 23) and HFrEF (*n* = 24), were enrolled at the Cardiology Unit of the University Hospital of Salerno. Sirt1 activity was measured in peripheral blood mononuclear cells (PBMCs). Angiotensin-Converting Enzyme 2 (ACE2) activity, Tumor Necrosis Factor-alpha (TNF-α) and Brain Natriuretic Peptide (BNP) levels were quantified in plasma. HFpEF showed lower Sirt1 and ACE2 activities than both HFmrEF and HFrEF (*p* < 0.0001), without difference compared to No HF controls. In HFmrEF and HFrEF a very strong correlation was found between Sirt1 activity and EF (r^2^ = 0.899 and r^2^ = 0.909, respectively), and between ACE2 activity and Sirt1 (r^2^ = 0.801 and r^2^ = 0.802, respectively). HFrEF showed the highest TNF-α levels without reaching statistical significance. Significant differences in BNP were found among the groups, with the highest levels in the HFrEF. Determining Sirt1 activity in PBMCs is useful to distinguish the HF patients’ phenotypes from each other, especially HFmrEF/HFrEF from HFpEF.

## 1. Introduction

Despite impressive advances in clinical/diagnostic tools and therapies, heart failure (HF) still represents a paramount public health problem being one of the most important causes of death and hospitalization worldwide [1]. As a syndrome, HF has multifactorial pathogenesis, and its diagnosis and management can be very demanding [2]. Determining the left ventricular ejection fraction (EF) is an essential diagnostic step [3].

The most recent guidelines of the European Society of Cardiology (ESC) consider three patient’s categories, Heart Failure (HF) with reduced Ejection Fraction (HFrEF; EF < 40%), HF with preserved ejection fraction (HFpEF; EF ≥ 50%), and HF with mid-range EF (between 40 and 49%) referred to as HFmrEF [4]. While there is an agreement that the categorization of HFrEF requires EF < 40%, the clinical overview of the patients with HFpEF has not been clearly established yet. The same ESC guidelines propose the measurement of the B-type natriuretic peptide (BNP) and/or N-terminal pro-BNP as an additional diagnostic criterion for HFmrEF and HFpEF, specifying that this cannot be useful to discriminate all three HF phenotypes because of its increase in other clinical conditions, including atrial fibrillation and renal failure, that compromise the interpretation of BNP and pro-BNP quantification [4].

Given the clinical implication, especially concerning comorbidities and therapy response, the correct characterization of the HF patients represents a crucial step for the management of this syndrome. One of the most important questions is if the HFmrEF represents a distinct phenotype or a transitional condition from HFrEF to HFpEF, or vice-versa [5]. However, doubtless, such a new category has been introduced to stimulate the research on these particular patients because there is an urgent need to identify new biomarkers and pharmacological targets helpful to choose the best therapy according to the different failing heart phenotypes.

Recent studies have investigated the possible role in the HF of sirtuins, a family of NAD+ dependent deacetylases, among which Sirtuin 1 (Sirt1) is the best-characterized member [6]. Sirt1 is involved in biological processes strongly related to HF, including oxidative stress, cell senescence, and energy production [7]. Moreover, it also plays a crucial role in angiotensin II-induced vascular remodeling [8] and inflammatory response modulating the expression of some cytokines [9]. For instance, tumor necrosis factor-alpha (TNF-α), which is involved in both central and peripheral manifestations of HF, has been found to increase in HFrEF when compared to HFpEF. Therefore, such a proinflammatory cytokine could be useful to separate the HF patients’ phenotypes from each other [9].

The overexpression of Sirt1 has been shown to favor the survival of cardiomyocytes, but to be also associated with cardiac hypertrophy and HF [10,11]. Indeed, both expression and activity levels of Sirt1 vary in the response of internal and external stimuli [12,13] and, following a hormetic mechanism, can be either advantageous or injurious [14]. However, until now, no data on Sirt1 activity according to the different HF phenotypes are available. Then, the present study aimed at evaluating in HFpEF, HFmrEF, and HFrEF patients whether the amount of Sirt1 activity was correlated with EF and other characteristics, including circulating TNF-α and angiotensin-converting enzyme 2 (ACE2) activity levels.

## 2. Materials and Methods

### 2.1. Study Population

Seventy patients with chronic HF in NYHA classes 2 and 3 were consecutively enrolled at the Cardiology Unit of the University Hospital of Salerno. Twenty-nine age-matched subjects without heart failure represented the control group (no HF controls). All the enrolled patients underwent a physical examination, blood chemistry tests, electrocardiographic and echocardiographic exams, and a 6-min walking test. Additionally, baseline demographic, clinical, echocardiographic, and functional data were collected on a predefined computerized datasheet. All subjects included in the study were in optimal medical therapy and managed according to ESC guidelines. All participants gave their written informed consent. The study was in accordance with the Declaration of Helsinki and its amendments and was approved by the local Ethical Committee (Comitato Etico Campania Sud Prot.n.4_r.p.s.o.).

### 2.2. Echocardiography

Transthoracic echocardiography (TTE) was performed following the ASE and ESC/EACVI recommendations using a Vivid E95 system with an M5S phased array and probe (GE Healthcare Vingmed Ultrasound AS, Horten, Norway). All echocardiographic images were digitally recorded. The left ventricular (LV) end-diastolic diameter (LVEDD) and the LV end-systolic diameter (LVESD) were measured using M-mode by the parasternal long-axis view; the LV volumes (LV end-diastolic volume, LVEDV, and LV end-systolic volume, LVESV) and the EF were calculated by the Simpson’s method using the apical 2-chamber and 4-chamber view. The LV diastolic function was characterized by the assessment of the ELV/e’LV ratio as a surrogate parameter of LV filling pressure. For the evaluation of the early-diastolic filling (E), the pulsed-wave Doppler sample volume was positioned at the tip of the tenting area of the mitral valve in the apical long-axis view. The mean of e’ was assessed in the basal inferoseptal and lateral LV region in the apical 4-chamber view using Tissue Doppler Imaging TDI. The left atrial (LA) volume index was calculated by biplane LA planimetry in the apical 2- and 4-chamber view. The right ventricular function resulted by the measurement of the tricuspid annular plane systolic excursion (TAPSE), and the pulmonary artery systolic pressure was estimated by tricuspid regurgitation velocity in the apical 4-chamber view and the right arterial pressure (RAP), derived from the inferior vena cava diameter and degree of respiratory collapse.

### 2.3. Six-Minute Walking Test

The 6-min walking test (6MWT) was performed according to the American Thoracic Society (ATS) guidelines [15].

The primary measurement was the total distance (meters, m) walked. The patients were instructed to walk up and down a corridor of 30 m, covering as much ground as possible in 6 min without running. Blood pressure was recorded at the end of the test, and pulse and oxygen saturation. The latter was measured by using a handheld pulse oximeter (G.i.ma. Spa, Milan, Italy) placed on the index finger of patients.

### 2.4. Blood Sampling and SIRT1 Activity

Blood samples were collected in fasting conditions in the BD Vacutainer^®^ containing sodium EDTA (BD, USA). The separation of plasma and peripheral blood mononuclear cells (PBMCs) was obtained by Ficoll density gradient centrifuged at 3000 rcf spin for 30 min at room temperature. Aliquots of plasma and PBMCs were frozen at −80 °C until further analysis.

To measure SIRT1 activity, nuclear extracts (10 μL) were isolated by PBMCs using a nuclear extraction kit (EpiGentek Group Inc., Farmingdale, NY, USA). Then, SIRT1 activity was determinate by a SIRT1/Sir2 Deacetylase Fluorometric Assay (CycLex, Ina, Nagano, Japan), following the manufacturer’s instructions. The values were reported as relative fluorescence/micrograms of protein (AU).

### 2.5. Circulating Angiotensin-Converting Enzyme 2 (ACE2) Activity and TNF-Alpha Measurement

The ACE2 activity was measured as previously described [16] using fluorogenic substrate 7-methoxycoumarin-4-yl) acetyl-Ala-Pro-Lys(2,4-dinitrophenyl)-OH (Mca-APK(Dnp)) Mca-Ala-Pro-Lys(Dnp)-OH (BioVision Inc., CA, USA). Briefly, plasma was diluted 1:10 in ACE2 reaction buffer containing protease inhibitors (10 μM Bestatin-hydrochloride, 10 μM Z-prolyl-prolinal, (Sigma, MO, USA), 5 μM Amastatin-hydrochloride, 10 μM Captopril in a buffer of 500 mM NaCI, 100 μM ZnCI2, and 75 mM TRIS HCI, pH 6.5). All chemicals were from Santa Cruz (CA, USA) if not stated otherwise. The reaction was performed at 37 °C in black 96-well microtiter plates in a total volume of 200 μL using a fluorescence plate reader (TECAN^®^ infinite 200 PRO) at an excitation wavelength of 320 nm and emission wavelength of 405 nm. Enzymatic activity was determined from a fluorescence rate increase over a 10–120 min time course, and the activity was reported as relative fluorescence units (RFU)/min.

The TNF-alpha evaluation was performed on plasma samples according to the manufacturer’s instruction (Diaclone, Human TNF-α ELISA Kit; #950.090.192).

The measurements of Sirt1 and ACE2 activities and TNF-alpha levels were performed in a blinded fashion. All data are expressed as the mean ± SD of three independent experiments.

### 2.6. Statistical Analysis

Data were analyzed using the SPSS (v 23.0) software package (SPSS Inc., Chicago, IL, USA). The Shapiro–Wilk test was used to assess the normal distribution of data. Differences between multiple groups were evaluated by the analysis of variance (ANOVA) with the Bonferroni post hoc test and are presented as mean ± SD. The χ2 test was used to compare categorical variables. A multiple linear analysis was used to investigate the relationship between variables when appropriate. To explore the correlation between variables, Spearman’s correlation (r) was used. The statistical significance was established at a *p*-value < 0.05.

The sample size was calculated from similar studies where Sirt1 activity determination in PBMCs was assessed in healthy individuals and HF patients (12, 17). We used an estimated standard deviation of 0.5 and the two-tailed alpha set at 0.05. An *n* = 9 per group was determined to provide sufficient power at 0.9 to detect a significant difference among groups.

Then a total of 99 subjects (29 No HF controls; 23 HFpEF; 23 HFmrEF; and 24 HFrEF) were included in the study.

## 3. Results

### 3.1. Study Population

The study population consisted of 99 subjects (66 M, 33 F; mean age 62.6 ± 9.4, and range 42–85) including 29 No HF controls and 70 HF patients (Table 1) belonging to the HFpEF (*n* = 23), HFmrEF (*n* = 23), and HFrEF (*n* = 24) categories defined according to the criteria of the ESC guidelines [4]. The main demographic and clinical characteristics of each group are reported in Table 1. Among the groups, no differences were found in age, gender, and body mass index (BMI). The echocardiographic findings confirmed the different types of the HF with the HFpEF group showing lower LVESV and LVEDV compared to both HmrEF and HFrEF (in both *p* < 0.0001), with an increasing trend between the groups through EF reduction.

From a functional point of view, all the HF groups showed a reduction in six-minute walking distance in comparison to the no HF controls (*p* < 0.0001) but no differences were found among the HF groups.

The HF groups were more affected by diabetes mellitus (*p* = 0.042) and used more diuretics (*p* < 0.0001), beta-blockers (*p* < 0.0001), ACE-inhibitors (*p* < 0.0001), and statins (*p* = 0.004) in respect to the no HF controls. No differences were found in comorbidities and therapy among the HF groups.

### 3.2. Sirt1 Activity

**Figure 1:**
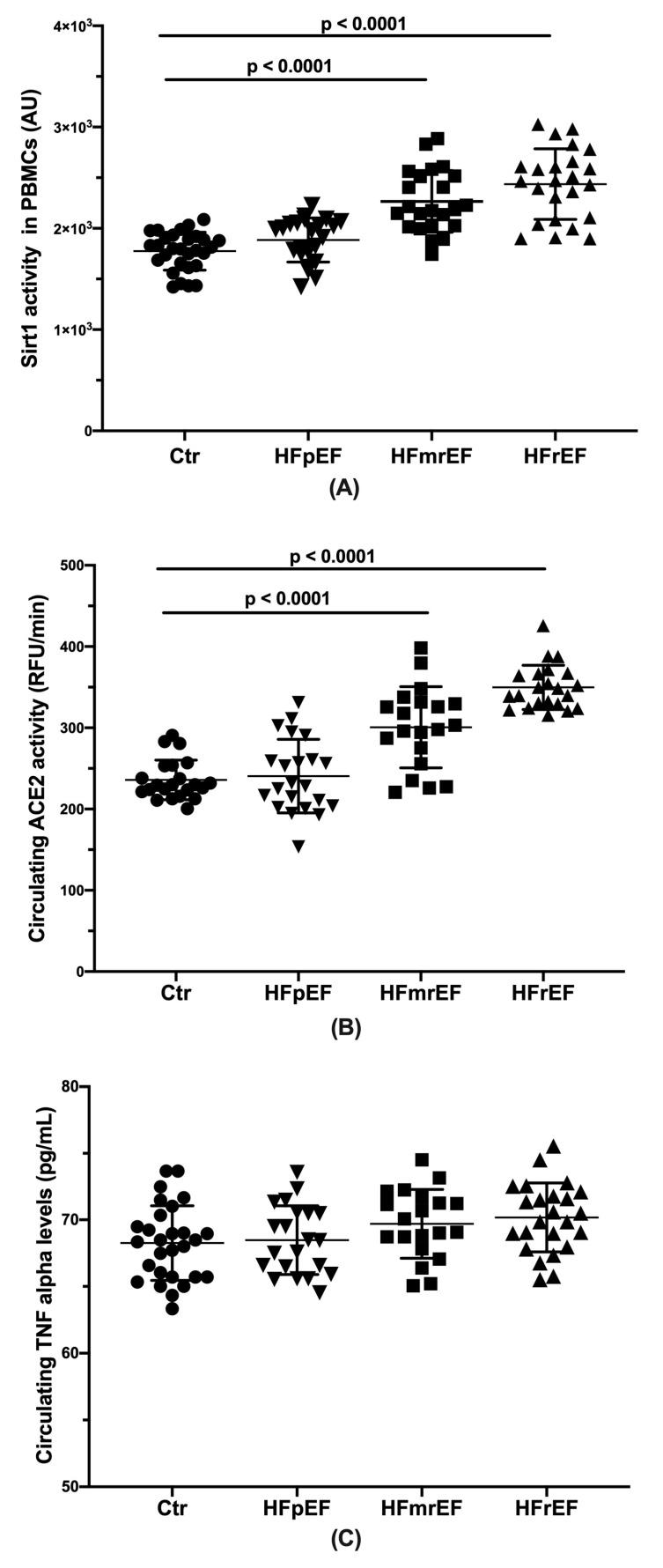
(**A**) Sirt1 activity levels by groups in the study population. Subjects with heart failure with preserved ejection fraction showed significant lower Sirt1 activity values than both subjects with heart failure with mid-range EF (HFmrEF) and those with heart failure with reduced EF (HFrEF) (*p* < 0.0001), without any difference compared to the no heart failure (HF) controls (Ctr). (**B**) Circulating ACE2 activity by groups in the study population. The HFpEF subjects showed significant lower ACE2 activity values than both HFmrEF and HFrEF (*p* < 0.0001), without any difference compared to the no HF controls (Ctr). (**C**) Circulating TNF-alpha levels by groups in the study population. An increasing trend of plasma TNF-alpha levels from the HFpEF through the HFmrEF to the HFrEF patients without reaching statistical significance was found.

The HFpEF subjects showed significant lower Sirt1 activity values than both the HFmrEF and the HFrEF (*p* < 0.0001), without any difference compared to the no HF controls (Figure 1A).

When a multivariate linear regression analysis was performed by using the EF as a dependent variable, after correction for the parameters statistically significant at the univariate analysis, the best predictors of EF were represented by Sirt1 activity (*p* < 0.0001; β = −0.019; 95%CI −0.023–0.014), and the use of beta-blockers (*p* = 0.001; β = −7.404; 95%CI −11.622–3.186).

The Sirt1 activity (used as dependent variable) was significantly associated to the HF groups (*p* = 0.003; β = −133.960; 95%CI −221.708–46.212). Then, because of the different characteristics of the HF in the three groups, to better explore the relationship between EF and Sirt1 activity, other multivariate regression analyses were performed. For each group, setting the EF as the dependent variable, the parameters statistically significant at the univariate analysis were identified, and then they were introduced in the multivariate analysis. In the no HF controls, the best predictors of EF were represented by the gender (*p* = 0.011; β = 4.539; 95%CI 1.132 7.946) and six-minute walking distance (*p* = 0.032; β = 0.067; 95%CI 0.006 0.127).

In the HFpEF group the EF was not associated to any variable. In the HFmrEF and HFrEF groups the best predictor of EF was represented only by the Sirt1 activity (for HFmrEF with *p* < 0.0001, β = −0.009, 95%CI −0.010–0.008 and for HFrEF with *p* < 0.0001; β = −0.011; 95%CI −0.013–0.010).

**Figure 2:**
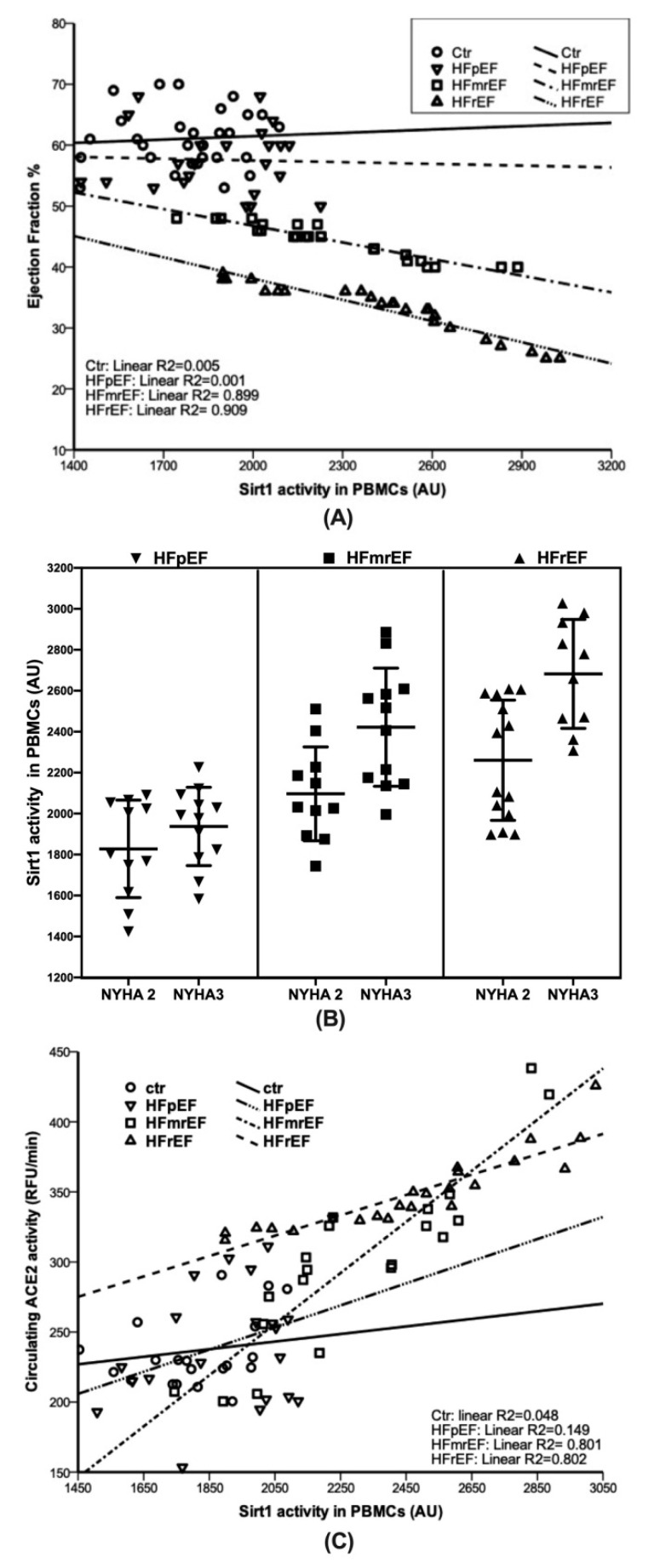
(**A**) Correlation between EF and Sirt 1 activity stratified by HF groups and no HF controls (Ctr). In the HFmrEF and HFrEF groups a very strong correlation was found between Sirt 1 activity levels and EF values (r^2^ = 0.899 and r^2^ = 0.909, respectively). Otherwise, in the HFpEF as in the no HF controls no correlation was found. (**B**) Association between NYHA classes and Sirt 1 activity stratified by HF groups. Sirt 1 activity represented the best predictor of NYHA classes in the HFrEF (*p* = 0.018, β = 1.006; 95%CI 1.001 1.010) and HFmrEF (*p* = 0.024; β = 1.005; 95%CI 1.001 1.009) but not in the HFpEF. (**C**) Correlation between ACE2 and Sirt 1 activities stratified by HF groups and no HF controls (Ctr). In the HFmrEF and HFrEF groups a very strong correlation was found between Sirt 1 activity and ACE2 activity levels (r^2^ = 0.801 and r^2^ = 0.802, respectively). Otherwise, in the HFpEF as in the no HF controls no correlation was found.

In Figure 2A, the correlation between Sirt1 activity and EF is pictured by groups, showing a very strong correlation between the two variables in the HFrEF (r^2^ = 0.909) and HFmrEF (r^2^ = 0.899), but not in the HFpEF (r^2^ = 0.001). Moreover, a logistic regression analysis with the NYHA classes, as the dependent variable, showed that Sirt 1 activity represented the best predictor in the HFrEF (*p* = 0.018, β = 1.006; 95%CI 1.001 1.010) and HFmrEF (*p* = 0.024; β = 1.005; 95%CI 1.001 1.009) but not in the HFpEF (Figure 2B). In particular, the higher NYHA class was significantly associated to the higher Sirt 1 activity levels in the HFrEF and HFmrEF.

These findings suggest the possible role of Sirt1 as a marker useful to distinguish the HF phenotypes.

### 3.3. Circulating Angiotensin-Converting Enzyme 2 (ACE2) Activity

The HFpEF subjects showed significant lower ACE2 activity values than both the HFmrEF and HFrEF (*p* < 0.0001) without any difference compared to the no HF controls (Figure 1B).

In Figure 2C, the correlation between the Sirt1 activity and ACE2 activity is pictured by groups, showing a very strong correlation between the two variables in the HFrEF (r^2^ = 0.802) and HFmrEF (r^2^ = 0.801), but not in the HFpEF (r^2^ = 0.149).

### 3.4. Circulating Tumor Necrosis Factor-Alpha (TNF-α) Levels

As shown in Figure 1C, no statistically significant differences in plasma levels of TNF-α were found among the groups. An increasing trend was observed from the HFpEF through HFmrEF to HFrEF. The No HF control subjects showed values closed to the HFpEF patients.

### 3.5. Circulating Brain Natriuretic Peptide (BNP) levels

At the univariate analysis, statistically significant differences in plasma levels of BNP were found among the groups, with the highest levels in the HFrEF group (Table 1). An increasing trend was observed from the HFpEF through HFmrEF to HFrEF. The no HF control subjects showed values close to the HFpEF patients. However, a multivariate linear regression analysis demonstrated no correlation between BNP and Sirt1 activity levels in all groups (for Ctr *p* = 0.434, r^2^ = 0.097, β = −0.026, 95%CI −0.104 0.052; for HFpEF *p* = 0.566, r^2^ = 0.024, β = −0.206, 95%CI −3.428 3.016; for HFmrEF *p* = 0.752, r^2^ = 0.025, β = −0.073; 95%CI −0.580 0.434; for HFrEF *p* = 0.388, r^2^ = 0.050, β = 0.126; 95%CI −0.175 0.427).

## 4. Discussion

The introduction in the recent ESC guidelines of the HFmrEF category [4] has given the impulse for investigations aiming at a better characterization of the patients suffering from HF. What emerges from the trials performed until now has highlighted that the clinical overview of the patients with HFpEF has not been adequately studied and, consequently, there are few effective treatments for them. Moreover, the HFmrEF represents a borderline group scarcely investigated, even less than HFpEF [3].

The processes and mechanisms involved in the cardiac failing phenotypes have not been exhaustively investigated, nonetheless elucidating the molecular card of the different HF patients might be of great help to better manage the disease and personalize the therapy.

Sirt1 represents a good candidate in this field because of its involvement in cardiac pathophysiology [17]. Indeed, historically Sirt1 has been recognized as an enzyme crucial to assure lifespan prolonging from yeasts to humans, and, in general, its decreased levels have been linked to endothelial dysfunction and the pathogenesis of metabolic and cardiovascular diseases [7,17,18,19]. Therefore, interventions aiming at increasing Sirt1 levels have been considered beneficial in aging and aging-associated diseases [20,21].

Sirt 1 expression and activity are often measured in PBMCs, which represent a model helpful to provide a comprehensive overview of the cellular system status together with measurement of circulating serum or plasma markers [22]. PBMCs are cells easy to isolate by a non-invasive and inexpensive method. This model has been used to study Sirt1 in several disorders such as diabetes mellitus [23], chronic obstructive pulmonary disease (COPD) [24,25], and in patients assuming a specific diet [26], or underwent cardiac rehabilitation [27].

Herein, we found that Sirt1 activity was much higher in PBMCs isolated from the HFrEF patients when compared to the HFmrEF and even more HFpEF. Importantly, the levels were so different making it possible to distinguish the HFmrEF/HFrEF from the HFpEF that, conversely, had Sirt1 activity very similar to the no HF control subjects. A very strong correlation between Sirt1 activity and EF values was found in the HFrEF (r^2^ = 0.909) and HFmrEF (r^2^ = 0.899), while no correlation in the HFpEF and no HF control subjects was observed. Additionally, the higher Sirt 1 activity levels were significantly associated with the higher NYHA class in the HFrEF and HFmrEF but not in the HFpEF.

Concerning the involvement of Sirt1 in cardiac remodeling, literature data show contrasting evidence. Some data report a relationship between increased Sirt1 levels and cardiac hypertrophy [28], while other data suggest that low-moderate Sirt1 overexpression has beneficial effects in contrasting fibrosis and hypertrophy [8]. Currently, several studies, while confirming a link between these conditions and Sirt1 increased levels, stressed the importance of a Sirt1 overexpression degree in determining beneficial or detrimental effects [14,29]. It is unsurprising when you consider that the intensity of caloric restriction and exercise training, interventions well recognized to activate Sirt1, makes the difference between their positive and negative effects [30,31,32]. Moreover, accumulating evidence has corroborated the idea that both expression and activity of Sirt1 vary in the response of internal and external stimuli, and the outcomes strongly depend on the cell type and condition [17]. Furthermore, understanding the role and the effects of Sirt1 in different contexts is essential, given the indubitable involvement of this enzyme in cardiovascular homeostasis and diseases [33]. In our opinion, the results of the present study go in such a direction. We found different Sirt1 activity levels with the highest value in the HFrEF patients, by measuring this parameter in HF patients classified (according to the recent ESC guidelines) in three different categories.

A possible explanation may be related to a link existing between Sirt1 and the renin angiotensin aldosterone system (RAAS) [34,35,36]. RAAS is one of the most important components of the so-called ‘neurohormonal’ system, designed to maintain cardiovascular homeostasis through a series of compensatory mechanisms. While this system is beneficial in the short term, its prolonged activation causes hemodynamic stress, cardiac and vessel structural modifications, and ultimately progression of HF, especially in HFrEF patients [33]. As a matter of fact, it is well known the better therapeutic response of HFrEF patients to beta-blockers, RAAS inhibitors, and angiotensin receptor–neprilysin inhibitors (ARNI), the latter licensed only in these subjects [33,37]. Furthermore, the lack of an optimal therapeutic response in HFpEF subjects represented one of the fundamental reasons to stimulate a better pathophysiology understanding of this HF phenotype [38].

It has been demonstrated both in vivo and in vitro that resveratrol, a polyphenol able to activate Sirt1, leads to a decrease of angiotensin II receptor AT1 through Sirt1 activation [34]. Another important result is that overexpression of Sirt1 exerts beneficial effects contrasting the angiotensin II-induced vascular remodeling and attenuating hypertension in mice [8]. In addition, an interesting study by Davis et al. performed in patients with Bartter’s/Gitelman’s (BS/GS) syndromes, who have a persistent RAAS activation with increased circulating levels of angiotensin II, showed that Sirt1 protein levels were higher in patients’ PBMCs than in those of healthy subjects [35]. Noteworthy, circulating AngII-degrading enzyme (ACE2) activity is much higher in the HFrEF in comparison with the HFpEF subjects. This finding indicates circulating ACE2 activity as a potential biomarker to differentiate these two cardiac failing phenotypes [39]. Moreover, Epelman et al. demonstrated that elevated plasma ACE2 activity was associated with greater severity of myocardial dysfunction, without a relationship between circulating ACE2 activity and markers of systemic inflammation [40]. On the contrary, Niethammer et al. found increased circulating levels of TNF-alpha in HFrEF in comparison to HFpEF patients and showed that such higher levels were negatively correlated to EF [9].

Here, we found an increasing trend of plasma TNF-alpha levels from the HFpEF through the HFmrEF to the HFrEF patients without reaching statistical significance.

Our results show that the HFrEF group had levels of ACE2 activity significantly higher than those measured in the HFmrEF and even more in the HFpEF subjects, with the latter showing no difference when compared with the No HF controls. Notably, a positive correlation between Sirt1 activity and ACE2 activity in the HFrEF (r^2^ = 0.802) and the HFmrEF (r^2^ = 0.801) but not in the HFpEF (r^2^ = 0.149) was found (Figure 2C).

Altogether these findings suggest a role for Sirt1 activity as a biomarker to distinguish the three HF phenotypes.

The highest Sirt1 activity in the HFrEF patients might reflect the high neurohormonal activation, including RAAS, which in turn characterizes systolic HF [33].

As already stated, the HFmrEF remains insufficiently characterized compared with the other groups. We found that the HFmrEF and HFrEF patients have similar Sirt1 levels, both much higher than the HFpEF and the correlation between Sirt1 activity and EF values in the HFmrEF was as relevant as in the HFrEF, even if less strong. However, the intermediate mean value of the Sirt1 activity found in the HFmrEF group (Figure 1A) seems to confirm the arising idea that the HFmrEF could represent an intermediate condition rather than a different HF category.

Sirt1 activity induced by pharmacological and non-pharmacological activators has been demonstrated to ameliorate the health status of HF patients [13,21,27]. Indeed, mild and moderate overexpression of Sirt1 might favor resistance to stress, thereby leading to cardiac positive outcomes, while further increased levels might be associated with cardiac damages [14]. Possibly, Sirt1 activity values, linked to beneficial effects, depending on the individual baseline levels, and their assessment could be useful in the management of the different HF patients.

In our opinion, the most important result of the present investigation is the existence, observed for the first time, of the relationship between the EF and Sirt1 activity with a very strong correlation between Sirt1 activity and EF in the HFmrEF and HFrEF. Of note, this correlation does not exist in the HFpEF patients.

This study is subject to some limitations. One of them is the lack of the cardiopulmonary stress test because it was performed not in all enrolled patients and no HF controls. Another limitation is the small number of patients included in each group.

As discussed, the high levels of Sirt1 might reflect an adaptive activation of the sympathetic system and RAAS characterizing the systolic HF. The relationship between Sirt1 and circulating ACE2 activity found in the HFrEF and HFmrEF but not in the HFpEF patients corroborates this hypothesis. Measuring factors other than ACE2 activity involved in the neurohormonal modulation could be helpful to better classify patients with HF.

Another limitation is the absence of Sirt1 activity levels definition in healthy subjects as a reference.

Undoubtedly, further and larger studies are necessary to measure such and other inflammatory parameters, other than TNF alpha, and verify whether they correlate with Sirt1 activity.

## 5. Conclusions

In our study, Sirt1 activity levels increased from the HFpEF through HFmrEF to the HFrEF.

Sirt1 activity in the HFmrEF, showing an average value between the HFrEF and HFpEF subjects, suggests the hypothesis that the HFmrEF represents an intermediate phenotype. This is supported by the finding of the strong correlation between Sirt1 activity and EF values observed also in HFmrEF patients.

The correlation between Sirt1 and ACE2 also reinforces the hypothesis that Sirt1 activity could be used as a biomarker to better differentiate the patients with different HF phenotypes, especially to separate HFmrEF/HFrEF from HFpEF.

Further studies with a larger sample size are needed to confirm or deny these results and clarify whether monitoring Sirt1 activity levels can effectively help the management of the patients suffering from HF. Moreover, more trials should also be performed to better understand the mechanisms underlining the HF phenotypes that could explain the different Sirt1 activation and to define the range of Sirt1 activity levels associated with beneficial effects.

## Figures and Tables

**Table 1:** Main characteristics of the study population stratified by No HF Controls and on the basis of HF type.

	Ctr (*n* = 29)	HFpEF (*n* = 23)	HFmrEF (*n* = 23)	HFrEF (*n* = 24)	*p*
Age, years	60.52 ± 8.91	63.87 ± 10.25	63.00 ± 9.16	63.50 ± 9.57	0.558
Sex, (M/F) n (%)	19/10 (65.5/34.5)	13/10 (56.5/43.5)	15/8 (65.2/34.8)	16/8 (66.7/33.3)	0.157
BMI, kg/m^2^	27.00 ± 3.14	27.89 ± 2.80	28.40 ± 3.80	28.07 ± 4.73	0.545
SBP, mmHg	126 ± 8 ^a^	123 ± 7 ^b^	121 ± 6 ^c^	106 ± 12	<0.0001
DBP, mmHg	81 ± 5 ^a^	80 ± 4 ^d^	79 ± 7 ^e^	72 ± 8	<0.0001
EF, %	61.07 ± 4.75 ^f,g^	57.61 ± 5.39 ^h^	44.35 ± 2.93 ^i^	33.03 ± 4.24	<0.0001
BNP, pg/mL	31.33 ± 14.00 ^f^	105.00 ± 64.42 ^b,j^	408.08 ± 55.5 ^i^	814.50 ± 193.83	<0.0001
LVESV, mL	32.56 ± 4.82 ^f^	44.30 ± 17.48 ^b,k^	72.63 ± 21.69 ^i^	122.17 ± 33.56	<0.0001
LVEDV, mL	84.83 ± 10.22 ^a^	103.17 ± 34.06 ^k^	131.68 ± 39.51 ^l^	171.25 ± 44.74	<0.0001
Cardiac Index, L/min/m^2^	2.94 ± 0.35	2.82 ± 0.47	2.74 ± 0.43	2.64 ± 0.36	0.059
SPAP, mmHg	28.41 ± 3.57 ^m,n^	40.00 ± 17.15	32.16 ± 5.83	36.33 ± 11.48	0.002
E/e’ ratio	6.72 ± 1.56 ^a,o^	12.85 ± 6.75	10.87 ± 2.61 ^p^	16.54 ± 8.03	<0.0001
Walking distance at 6′, m	522.69 ± 26.63 ^q^	387.30 ± 56.14	406.65 ± 49.14	408.54 ± 73.69	<0.0001
Walking distance at 6′ ≥ 350 m, n (%)	29 (100.0)	19 (82.6)	18 (78.3)	18 (75.0)	0.049
CKD, (yes) n (%)	0 (0)	5 (22.2)	6 (26.7)	8 (35)	0.116
Hypertension, (yes) n (%)	10 (34.5)	16 (69.6)	14 (60.9)	14 (58.3)	0.063
Dyslipidaemia, (yes) n (%)	7 (24.1)	12 (52.2)	12 (52.2)	14 (58.3)	0.051
Smoking, (yes) n (%)	8 (27.6)	5 (21.7)	9 (39.1)	6 (25.0)	0.582
Diabetes Mellitus, (yes) n (%)	2 (6.9)	8 (34.8)	6 (26.1)	9 (37.5)	0.042
COPD, (yes) n (%)	3 (10.3)	5 (21.7)	4 (17.4)	6 (25.0)	0.541
Prior MI, (yes) n (%)	0 (0)	4 (26.7)	8 (44.4)	12 (50.0)	0.415
HF etiology, (yes) n (%)					
Ischemic cardiomyopathy	0 (0)	5 (21.7)	11 (47.8)	19 (79.2)	0.004
Valvular cardiomyopathy	0 (0)	6 (26.1)	5 (21.7)	2 (8.3)	0.083
Hypertensive cardiomyopathy	0 (0)	5 (21.7)	4 (17.4)	1 (4.2)	0.329
Primary cardiomyopathy	0 (0)	7 (30.4)	3 (13)	2 (8.3)	0.195
Diuretics, (yes) n (%)	1 (3.4)	8 (34.8)	27 (73.9)	20 (83.3)	<0.0001
Beta-blockers, (yes) n (%)	3 (10.3)	22 (95.7)	21 (91.3)	22 (91.7)	<0.0001
ACE-inhibitors, (yes) n (%)	4 (13.8)	14 (60.9)	15 (65.2)	15 (62.5)	<0.0001
ARBs, (yes) n (%)	3 (10.3)	7 (30.4)	7 (30.4)	3 (12.5)	0.132
Statins, (yes) n (%)	8 (27.6)	15 (65.2)	16 (69.6)	16 (66.7)	0.004

Ctr, No Heart Failure Controls; HFpEF, Heart Failure with preserved Ejection Fraction; HFmrEF, Heart failure with mid-range Ejection Fraction; HFrEF, Heart Failure with reduced Ejection Fraction; BMI, Body Mass Index; SBP, Systolic Blood Pressure; DBP, Diastolic Blood Pressure; EF, Ejection Fraction; BNP, Brain Natriuretic Peptide; LVESV, Left Ventricle End Systolic Volume; LVEDV, Left Ventricle End Diastolic Volume; SPAP, Systolic Pulmonary Artery Pressure; CKD, Chronic Kidney Disease; COPD, Chronic Obstructive Pulmonary Disease; MI, Myocardial Infarction; ARBs, Angiotensin Receptor Blockers. ^a^ Ctr vs. HFrEF *p* < 0.0001; ^b^ HFpEF vs. HFrEF *p* < 0.0001; ^c^ HFmrEF vs. HFrEF *p* = 0.002; ^d^ HFpEF vs. HFrEF *p* = 0.001; ^e^ HFmrEF vs. HFrEF *p* = 0.035; ^f^ Ctr vs. HFmrEF and HFrEF group *p* < 0.0001; ^g^ Ctr vs. HFpEF *p* = 0.038; ^h^ HFpEF vs. HFmrEF and HFrEF group *p* < 0.0001; ^i^ HFmrEF vs. HFrEF *p* < 0.0001; ^j^ HFpEF vs. HFmrEF *p* = 0.010; ^k^ HFpEF vs. HFmrEF *p* = 0.002; ^l^ HFmrEF vs. HFrEF *p* = 0.001; ^m^ Ctr vs. HFpEF *p* = 0.002; ^n^ Ctr vs. HFrEF *p* = 0.028; ^o^ Ctr vs. HFpEF *p* = 0.003; ^p^ HFmrEF vs. HFrEF *p* = 0.004; ^q^ Ctr vs. all other groups *p* < 0.0001.
